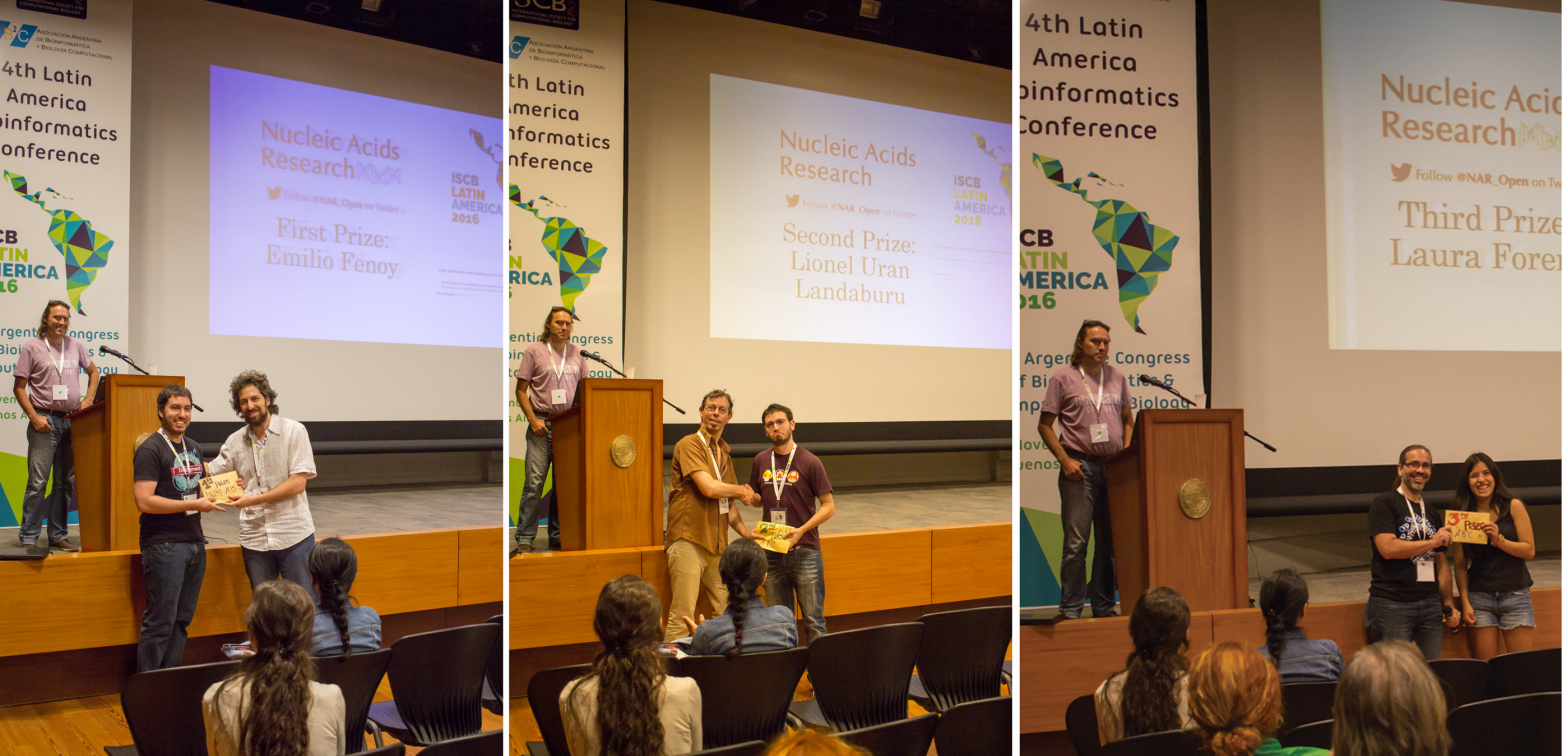# Editorial: NAR Awards 2016

**DOI:** 10.1093/nar/gkw1168

**Published:** 2016-12-10

For the eleventh year running, NAR and Oxford Journals have provided sponsorship and awarded travel bursaries and prizes to students and junior scientists in recognition of their outstanding achievements. This year, sponsorship and prizes were given at 9 different meetings. A list of the awards is presented below. Our warm congratulations go to the prize winners.

**Student New Investigator Poster Session**

**Environmental Mutagenesis and Genomics Society**

Kansas City, MO, USA

September 24–28, 2016

**Poster Prizes**

**SifrARN meeting, French Society for Biochemistry and Molecular Biology**

Toulouse, France

March 8–10, 2016

**Jonathan Jagodnik** (Institut de Biologie Physico-Chimique IBPC, CNRS UMR8261 - Expression Génétique Microbienne)

**International Society for Computational Biology Student Council Meeting**

Orlando, FL, USA

July 8–12, 2016

**Urszula Czerwinska** (Institute Curie, Paris Descartes, France)

**FASEB meeting on Post-transcriptional control of gene expression: Mechanisms of RNA decay**

Lisbon, Portugal

July 10–15, 2016

**Jeffrey Mugridge** (University of California – San Francisco, CA, USA)

Cap recognition and catalytic activation of the Dcp1/Dcp2 mRNA decapping complex

**International Biennial Conference on ‘LARPs’**

Stockton House, Wiltshire, UK

September 14–17, 2016

**Roni Lahr** (University of Pittsburgh, PA, USA)

LARP1 interacts directly with 5’UTRs encoding translation machinery

**International Retroviral NucleoCapsid and Assembly Symposium**

Montpellier, France

September 18–21, 2016

**Assia Mouhand** (Laboratoire de Cristallographie et RMN biologiques, Université Paris Descartes, Paris, France)

The C-terminal domain of Gag: multiple interactions and role for the assembly and budding of HIV-1

**RNA Biochemistry of the German Society for Molecular Biology and Biochemistry Meeting**

Bonn, Germany

October 6–9, 2016

**Indira Memet** (Institute for Molecular Biology, Medical Department, Georg-August Universität)

Interactions and functions of the NFκB-repressing factor in human ribosome biogenesis

**Elke Duchardt-Ferner** (Institute for Molecular Biosciences and Center of Biomolecular Magnetic Resonance (BMRZ), Goethe University, Frankfurt, Germany)

Structure and Folding of Tetramethylrhodamine Binding RNA Aptamers

**Oligonucleotide Therapeutics Society**

Montreal, Quebec, Canada

September 25–28, 2016

**Yazan Abbas** (McGill University, Montreal, Quebec, Canada)

RNA binding by Interferon Induced Proteins with Tetratricopeptide Repeats

**Hala Abou Assi** (McGill University, Montreal, Quebec, Canada)

Trapping G-quadruplex/i-Motif Intermediates of Human Telomeric DNA

**Annabelle Biscans** (University of Massachusetts Medical School, MA, USA)

Synthesis and Optimization of Docosahexaenoic Acid Conjugated Oligonucleotides for siRNA applications

**Jovanka Bogojeski** (McGill University, Montreal, Quebec, Canada)

Novel Tailed siRNA containing 2′-N-Acyl-L-Phenylalanine Sugar Modifications Self-Delivers to Reduce DRR Expression in Malignant Glioblastomas

**Amy E. Brinegar** (Baylor College of Medicine, Houston, TX, USA)

Redirected splicing by morpholinos to determine function on calcineurin splicing in skeletal muscle development

**Andrew Coles** (University of Massachusetts Medical School, MA, USA)

Polyunsaturated Fatty Acid Conjugated hsiRNAs Enables Systemic Delivery and Efficacy in Mouse Tissues

**Laura Croft** (Queensland University of Technology, Brisbane, Australia)

Targeting DNA damage response pathways with antisense oligonucleotides

**Suzan Hammond** (University of Oxford, Oxford, UK)

Peptide conjugated antisense oligonucleotides cross the blood brain barrier to enhance their therapeutic efficacy for spinal muscular atrophy

**Masahiko Horiba** (Osaka University, Osaka, Japan)

Synthesis of 2’-O,4’-C-Spirocyclopropylene Bridged Nucleic Acids (scpBNA) Bearing Purine Bases

**Zachary Kartje** (Southern Illinois University, Carbondale, IL, USA)

Nucleotide Substitutions Tune CRISPR/Cas9 Cleavage Activity

**Kassie Manning** (Baylor College of Medicine, Houston, TX, USA)

Evaluating antisense therapy for the treatment of myotonic dystrophy

**Louise Usher** (University of Westminster, London, UK)

Translationally relevant assays for documenting the mechanism of action of oligonucleotide therapeutics in the clinic

**International Society for Computational Biology Latin American Conference**

Buenos Aires, Argentina

November 21–23, 2016

**Emilio Fenoy** (Universidad Nacional de San Martin, Argentina)

NetPhosPan: a pan specific predictor for phosphorylation site predictions

**Lionel Uran Landaburu** (Instituto de Investigaciones Biotecnológicas, UNSAM - CONICET, Agentina)

Updates to the TDR Targets chemogenomics database

**Laura Forero** (Universidad de los Andes, Colombia)

Identification of Bacteriophage crAssphage Through Hidden Markov Models